# Supplementary material for: Carbon pricing and system reliability impacts on pathways to universal electricity access in Africa
Source: Nat Commun. 2024 May 16;15:4172. doi: 10.1038/s41467-024-48450-7 (PMC11099103; doi:10.1038/s41467-024-48450-7)
Supplement: Supplementary file 1 — Supplementary information [file 41467_2024_48450_MOESM1_ESM.pdf]

Supplementary Information

Carbon pricing and system reliability impacts on pathways to universal electricity access in Africa

Hamish Beath [1,2], Shivika Mittal [2,3], Sheridan Few [2,4], Benedict Winchester [2,5], Philip Sandwell[1,2], Christos N. Markides [5], Jenny Nelson [1,2], and Ajay Gambhir [2],

1. Department of Physics, Imperial College London, London SW7 2AZ, UK
2. Grantham Institute – Climate Change and the Environment, Imperial College London, London SW7 2AZ, UK
3. CICERO Center for International Climate Research, Oslo, Norway
4. Sustainability Research Institute, School of Earth and Environment, University of Leeds, Leeds LS2 9JT, UK
5. Clean Energy Processes (CEP) Laboratory, Department of Chemical Engineering, Imperial College London

Supplementary Note 1: Solar Data Inputs

The Renewables.ninja model requires tilt and orientation (azimuth towards north) as well as coordinates for a given location; the model has a spatial resolution of 50km^2 and provides outputs on an annual basis[1]. For panel tilt, we used the value of the latitude (multiplied by -1 for countries in the Southern Hemisphere) and with a minimum value of 10 degrees reflecting the need for some angle for rainwater cleaning. For the orientation, we assume panels are placed horizontally tilting towards the equator, either Northwards in the Southern Hemisphere or Southwards in the Northern Hemisphere. For the latitude and longitude for each country; the country centroids were used as defined in the list provided in Google Developer Tools[2].

Supplementary Table 1

Describing the population and population without access in 2020 and expected in 2030 and 2035 under the baseline scenario.

Year	Population Total Without Access	Net Change in No Access	Total population
2020	505 million		1.10 billion
2025	478 million	-27 million	1.21 billion
2030	472 million	-6 million	1.34 billion
2035	471 million	-1 million	1.46 billion

Supplementary Note 2: Ref_late scenario

The reduction in the cost of solar PV increases its competitiveness compared to other technologies used for electricity access in the Ref_late scenario. The results show a significant increase in the share of PV-based off-grid systems in Tier 2 in the Ref_late scenario (51% in 2035) compared to the Ref_central scenario (36% in 2030). The increase in the share of off-grid PV-based systems meeting the electricity demand is lower at other Tiers of access: 6% and 7% higher in *Ref_late* compared to the *Ref_central* scenario for Tiers 3 and 4. The results suggest that the timing of the universal access target and demand level have a significant influence on the cost competitiveness of off-grid PV compared to diesel or grid extension.

Supplementary Note 3: Rel_grid_all scenario

For the *Ref_central* scenario, the off-grid systems are sized to meet 'high' proportions (90%) of the total electricity demand. In the *Rel_grid_all* scenario, we look at the cost-optimal outcome for Tiers 2 to 4 when the off-grid systems are sized to meet the same assumed reliability as the grid in rural areas in each country (Table 1, Methods). We find a decline in the share of the population connected via the grid, reducing from 29 to 22% at Tier 2, 49 to 29% at Tier 3 and 63 to 34% at Tier 4 compared to the *Ref_central* scenario. At Tier 2, this reduction in grid share is entirely met by an increased share of off-grid PV systems; and the share of diesel also falls. At Tiers 3 and 4, shares of both off-grid diesel and PV systems increase. At Tier 3, the majority of the fall in grid share is met by PV systems, and at Tier 4 the majority is met by diesel generators. These results highlight the

Supplementary Figure 1

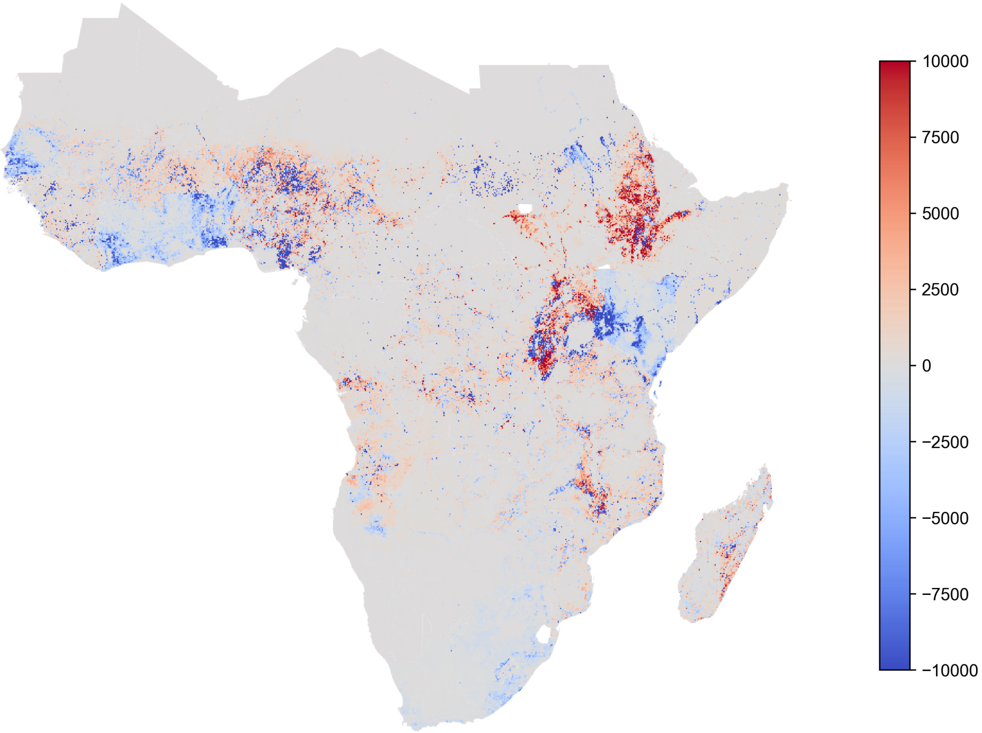

Spatial distribution at $10km^2$ resolution showing population without access in 2020 and 2030. Areas coloured red represent areas where the population without access is expected to increase, with blue being areas where the population is expected to decrease. Countries such as Ghana, Kenya, South Africa, and Ivory Coast are expected to reach universal access which can be seen in areas of blue. The results indicate that parts of the Sahel and East African countries such as Ethiopia and Uganda are expected to see the greatest increase. Whilst other approaches factor in stated policies, our approach considers historical trends in changes in access and anticipated population growth only (see methods).

importance of sizing off-grid systems (PV or diesel) in relation to the percentage of energy demand met for least-cost electricity access pathways when compared to grids, which may have low levels of reliability.

Supplementary Note 4: Applying a reliability subsidy

In our analysis, we have applied a reliability penalty for units of unmet electricity demand. Depending on what may be more feasible for a given country context, a reliability subsidy may be preferable instead. This could be applied in such a way that it would be mathematically identical to applying the reliability penalty, and therefore have the same impact on the change of shares of technology used when compared to the *Ref_central* scenarios.

The approach to have the same impact as a penalty is to simply apply a subsidy to units of electricity supplied by off-grid (PV or Diesel) systems above the assumed reliability of the rural grid, rather than to apply a penalty to all units not met (see Methods). This yields the same results, as is shown in Figure below.

Supplementary Figure 2

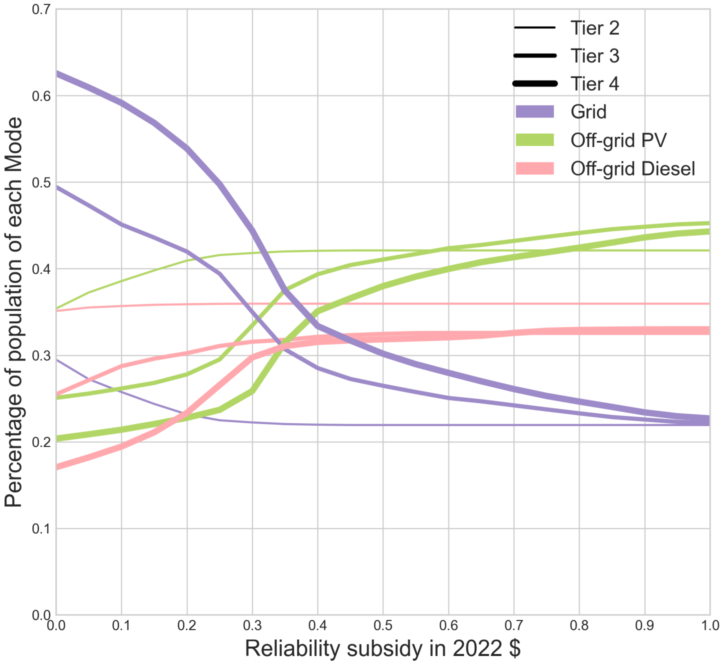

Figure showing the shares of the population served by each technology category (grid, off-grid PV, off-grid diesel) at electricity demand tiers 2-4, when applying a subsidy (as described above) to units of demand met by off-grid systems above the grid's reliability in a given country. Technology category is determined by line colour, and demand tier by line thickness. The trends shown in this figure are identical to those shown in Figure 3c; as the mathematical impact is the same as applying a penalty. $ are 2022 US Dollars.

Supplementary Note 5: Component cost reductions

We assume only a modest decline in combined battery, PV panel and inverter cost reductions due to uncertainty in the global economy regarding supply chains, inflation and global conflict/sanctions. Cost reductions may in reality be more marked, following historical trends. This would more greatly favour the deployment of off-grid.

Supplementary Table 2

Model inputs used for technical specification of off-grid systems. C-rate is the charge rate for the battery, AC refers to alternating current, and DC to direct current. LFP refers to Lithium iron phosphate battery chemistry.

Item	Value	Unit	Reference	Notes
Battery Depth of Discharge	70	%	[3]	LFP battery chemistry
Battery Leakage	0.004	% per hour	[4]	LFP battery chemistry
Battery Cycle lifetime	2500	Cycles	[5]	LFP battery chemistry
Battery Roundtrip efficiency	86	%	[4]	
Battery Lifetime loss	80	%	[5]	
Battery C-rate in	1			
Battery C-rate out	1			
Battery unit size (stand alone)	0.2	kWh		
Battery unit size (mini-grid)	1	kWh		
Transmission efficiency AC	92	%	[6]	
Transmission efficiency DC	96	%	[6]	
DC to AC conversion	97	%	[7]	
DC to DC conversion	95	%	[6]	
AC to DC conversion	90	%	[8]	
AC to AC conversion	98	%	[7]	
PV Inverter lifetime	10	Years		
Battery Inverter lifetime	15	Years		
Diesel consumption	0.28	Litres/kilowatt/hour	[9]	
Diesel minimum size	1	kW		
Diesel minimum load factor	0.35	%		
Solar PV Lifetime	20	Years		
Solar PV unit size (stand alone)	0.05	kilowatt peak		
Solar PV unit size (mini-grid)	0.3	kilowatt peak		

Supplementary Table 3

Model inputs used for the economic and greenhouse gas assessment of off-grid systems. O& M refers to operation and maintenance, CAPEX to capital expenditure and kgCO2/kWp is kilograms of carbon dioxide per kilowatt peak of installed capacity. kWp refers to kilowatt peak, kW to kilowatts and kWh to kilowatt hours. $ are 2022 US Dollars.

Item	Cost/Value	Unit	Ref	GHGs	Unit	Ref
Solar PV Panels	400	$/kWp	[10]	790*	kgCO2/kWp	[11]
Solar PV Cost Decrease	3	% per year (p.a.)				
Solar PV O&M	1	% of CAPEX p.a	[9]			
PV Inverter	163	$/kW	[10]	124*	kgCO2/kWp	[12]
PV Inverter Cost Decrease	3	% p.a				
Battery Storage	300	$/kWh	[10]	110	kgCO2/kWh	[13]
Battery Storage Cost Decrease	7.5	% p.a	[14]			
Battery O&M	1	% of CAPEX p.a	[9]			
Balance of System	100	$/kWh	[10]	134	kgCO2/kWp	[12]
Diesel Generator	400	$/kWp	[15]	476	kgCO2/kW	[16]
Diesel Fuel	See table A.5			2.67	kgCO2/litre	[17]
Diesel O&M	10	% of CAPEX p.a	[9]			
Meter & Distribution Cost	125	$ per household	[18]			

*Based on manufacture in China.

Supplementary Table 4

Non-country specific model inputs used for the least-cost electrification modelling, km refers to kilometres, sq km is kilometres squared, kg CO_2-eq is kilograms of carbon dioxide equivalent. $ are 2022 US Dollars.

Item	Value	Unit	Source
Grid Cost	16000	$/km	[19]
Meter Cost	90	$ per household	[19]
Install cost	250	$ per household	[10]
Discount Rate	8	%	[19]
Density Threshold	200	people per sq km	[10]
Grid emissions factor	3000	kg CO_2-eq per km	
Grid Transmission Lifetime	20	Years	
PV System Lifetime	20	Years	

Supplementary Table 5

Percentile values of carbon prices from the Intergovernmental Panel on Climate Change 6th Assessment report for scenarios limiting warming to 1.5-degrees celsius (C1 and C2) in 2022 US Dollars per tonne of carbon dioxide equivalent[44]

Percentile	2020	2025	2030	2035
10	0.00	18.44	20.75	98.91
20	0.00	28.28	44.05	135.54
30	0.32	39.80	67.94	183.78
40	2.45	64.96	98.30	213.16
**50**	**3.69**	**89.11**	**148.01**	**272.51**
60	5.86	120.65	191.40	320.46
70	9.53	161.66	248.40	364.02
80	10.91	200.48	288.77	445.20
90	13.94	271.80	381.10	694.04

Supplementary Table 6

Country-level data used for the least-cost electricity access modelling. Diesel prices are taken from World Bank data for the most recent value for each country[20]. Rural electricity demand met is based on data from the World Bank Enterprise Survey[21]. Rural household size by country is taken from Global Data Lab database[22]. Grid capacity costs and capacity factors are calculated using estimated shares from each country (see Methods) with cost and capacity data by country from Allington (2022)[23]. Grid emissions intensity data is taken from various sources[24–26]. kWh is kilowatt hours, kgCO2 is kilograms of carbon dioxide and $ are 2022 US Dollars.

Country	Diesel Prices	Rural Grid Electricity Demand Met	Household Size	Grid Capacity Cost ($/kWh)	Grid Capacity Factor	Grid Emissions Intensity (kgCO2/kWh)
Angola	0.82	66%	5.94	2129	0.61	1.48
Benin	0.72	61%	8.3	1241	0.82	0.58
Botswana	0.67	73%	5.63	2500	0.85	1.03
Burkina Faso	0.86	71%	7.2	1447	0.75	0.58
Burundi	1.14	64%	5.94	2128	0.54	0.41
Cameroon	0.94	66%	8.66	2018	0.69	0.66
Central African Republic	1.4	43%	6.96	2452	0.70	0.19
Chad	0.85	70%	7.84	1269	0.82	0.75
Cote d'Ivoire	0.93	72%	7.02	1642	0.67	0.58
Djibouti	0.61	66%	6.18	1300	0.80	0.75
DRC	1.2	66%	6.21	2475	0.34	1.00
Equatorial Guinea	0.94	66%	8.45	1651	0.62	0.63
Eritrea	0.64	66%	6.37	1304	0.78	0.92
Ethiopia	0.64	68%	5.96	2460	0.40	0.12
Gabon	1.2	66%	6.11	1754	0.71	0.95
Gambia	1.06	58%	15.6	1300	0.80	0.71
Ghana	0.85	66%	7.97	1760	0.73	0.58
Guinea	0.9	73%	7.82	1980	0.60	0.75
Guinea-Bissau	0.9	62%	9.42	1300	0.80	0.75
Kenya	0.82	72%	5.83	2987	0.65	0.50
Lesotho	0.73	73%	5.5	2500	0.69	1.00
Liberia	0.83	70%	6.74	1912	0.70	0.68
Madagascar	0.93	73%	5.83	2068	0.76	0.88
Malawi	1.14	71%	5.49	2199	0.59	0.49
Mali	0.98	72%	7.41	2068	0.63	0.58
Mauritania	1.09	73%	7.95	1323	0.73	0.75
Mozambique	0.6	74%	5.46	2253	0.72	1.03
Namibia	0.74	75%	6.94	2443	0.60	1.03
Niger	0.88	59%	7.46	1691	0.80	0.58
Nigeria	0.64	23%	7.28	1461	0.74	0.58
Republic of Congo	1.2	66%	5.66	1504	0.76	0.66
Rwanda	1.13	75%	5.16	1748	0.65	0.77
Senegal	0.97	74%	12	1535	0.78	0.58
Sierra Leone	1.08	58%	7.23	2137	0.49	0.49
Somalia	0.61	66%	7.06	1300	0.80	0.75
South Africa	0.9	73%	6.09	2604	0.81	1.03
South Sudan	0.61	66%	6.76	1301	0.79	0.89
Sudan	0.61	74%	7.25	2020	0.58	0.21
Tanzania	0.8	67%	7.15	1721	0.69	0.53
Togo	0.71	73%	5.15	1632	0.64	0.58
Uganda	0.79	66%	6.28	2431	0.54	0.57
Zambia	1.16	55%	6.62	2441	0.66	1.03
Zimbabwe	1.18	72%	5.33	2488	0.77	1.03

Supplementary Table 7

Showing the substitutions used for electricity demand, diesel prices or CLOVER energy system modelling outputs when the required data inputs were unavailable. Country substitutions were selected based on countries with the most similar socio-economic profile in close geographical proximity and with data availability

Country	Electricity Demand	Diesel	CLOVER Modelling
Angola			
Benin			
Botswana			
Burkina Faso	Benin		
Burundi	DRC		
Cameroon			
Central African Republic	DRC		
Chad			Niger
Cote d'Ivoire			
Djibouti			Sudan
DRC			
Equatorial Guinea			Cameroon
Eritrea			Ethiopia
Ethiopia			
Gabon			Republic of Congo
Gambia	Senegal		
Ghana			
Guinea	Senegal		
Guinea-Bissau	Senegal	Guinea	
Kenya			
Lesotho	Botswana		
Liberia	Ghana		
Madagascar	DRC		
Malawi	Zambia		
Mali	Niger		
Mauritania	Senegal		
Mozambique			
Namibia			
Niger			
Nigeria			
Republic of Congo		DRC	
Rwanda	Tanzania		
Senegal			
Sierra Leone	Senegal		
Somalia			Sudan
South Africa			
South Sudan			Sudan
Sudan			
Tanzania			
Togo			
Uganda	Tanzania		
Zambia	Zimbabwe		
Zimbabwe			

Supplementary Methods

The model used in this paper is written in Python and processes inputs and provides outputs in CSV files[27]. The geographical area of study is split into 'cells', each with its own unique ID number and location information such as the country or district it sits within and the coordinates of the cell boundaries. The model is designed with an annual temporal resolution, and a flexible spatial resolution (depending on input data) and can be used to assess what the least cost mode of electrification is in each gridded cell under different scenarios. The model is designed to compare the cost of grid extension with off-grid modes of access: stand-alone systems and mini-grids. These off-grid modes could be powered by any generation means, however; for the usage in this paper it is is configured to compare diesel and PV-based systems and the description below will cover those systems only.

The model requires a range of inputs and geospatial data layers before completing the cost-minimisation exercise. Firstly, the model requires a geospatial layer that describes the number of people who will gain access per year in each map cell (see methods). This can be created by using relevant geospatial data sets such as population, population without access and population growth. The population inputs - a matrix of numbers of people gaining access by year and by map cell - define the simulation period covered. Using household size, which may vary by country (see Supplementary Table 6), region or district, allows the model to know the number of households that need access in each map cell per year of the simulation. Additionally, the model requires a vector layer detailing the existence of national grid infrastructure and energy demand estimates per household. Cost inputs for each technology and system type are also required (Supplementary Tables 3, 4 and 6, Methods).

National Grid Costs

Grid extension costs, for a given cell ϕ; are defined by the centroid of the cell's distance from the nearest grid line G^{dist} (in km), multiplied by the grid extension cost, C^{km} (in \$ per km),

$$C_G^{ext}(\phi) = C^{km}\left(G^{dist}(\phi)\right) \tag{5}$$

Grid capacity costs, C_G^{cap} (\$/kW) for a given cell ϕ are calculated using an estimation of the additional capacity required for each cell $G^{cap}(\phi)$ (in kW). The additional capacity per cell $G^{cap}(\phi)$ is calculated using the weighted average grid capacity factor G_y (%) for the country κ of the given cell (ϕ), and the average hourly electricity demand, E^L (in kWh), in the final year, N, in the selected cell ϕ, taking into account the assumed reliability of the national grid in rural areas, in each country, $G^R(\kappa)$. The additional grid capacity that would be required in each cell $G^{cap}(\phi)$ is therefore given by

$$G^{cap}(\phi) = \left(\frac{1}{G_y(\kappa)}\right)\left(E_N^L(\phi)\right)\left(G^R(\kappa)\right) \tag{6}$$

The grid capacity investment requirements (in 2022 USD) for the cell, $C_G^{cap}(\phi)$, are given by

$$C_G^{cap}(\phi) = \left(G^{cap}(\phi)\right)C_G^I(\kappa) \tag{7}$$

where $C_G^I(\kappa)$ is the assumed grid capacity cost (in \$/kW) for the country the cell is within. The total grid costs for each cell $C_G^{tot}(\phi)$ are the sum of the above costs in all years, each discounted to the year of construction n, at discount rate r:

$$C_G^{tot}(\phi) = \sum_{n=1}^{N} \frac{\left(C_G^{ext}(\phi) + C_G^{cap}(\phi)\right)}{(1+r)^n} \tag{8}$$

the year of construction is determined by the population inputs and is covered in more detail below. Considering all map cells in the simulation, the model outputs a column vector of total costs, $C_G^{tot}(\Phi)$ to be used later in the model.

National Grid Emissions

The emissions from each source are used to provide emissions totals, for each scenario. They are also used to calculate carbon tax amounts added for scenarios where carbon pricing is included. Emissions are tracked for each map cell, ϕ, and by year, n. The total grid emissions by cell (kgCO_2-eq), and for a given year, $\xi_{G,n}(\phi)$, comprise firstly, embedded emissions in grid infrastructure for the cell and year. The year the grid is extended to a given cell ϕ, is determined by the user-defined population model (see Methods), which provides the year that the first household within a non-connected cell gains access. The model stores a binary matrix for all cells and years, based on the population model, with 1, denoting the year of the first connection and thus the building of the required infrastructure, $\Theta_n(\phi)$. Grid extension emissions are split annually over the asset lifetime, α^{ext} (in years, Supplementary Table 4), for every year following the year of construction (when $\Theta_n(\phi)$ = 1) until the end of the project lifetime, and are accounted for using the following

$$\xi_{G,n}^{ext}(\phi) = \begin{cases} \sum_{x=n}^{N} \frac{\xi^{km}\left(G^{dist}(\phi)\right)}{\alpha^{ext}} & \text{if } \Theta_n(\phi) = 1 \\ 0 & \text{otherwise} \end{cases} \tag{9}$$

where ξ^{km} is the embedded emissions (kgCO_2-eq) per km of grid extension, $G^{dist}(\phi)$ is the distance from the grid of the given cell ϕ, x is any year between the construction year n, and the end of the simulation N. The second component of national grid emissions is from electricity generation ξ_G^{Gen}, which is dependent on the generation mix for the country κ; and the associated emissions intensity of electricity generated, $\xi_G^O(\kappa)$, (in kgCO_2-eq/kWh) and the electricity demand E^L (in kWh) in a given year, n:

$$\xi_{G,n}^{Gen}(\phi) = \xi_G^O(\kappa)\left(E_n^L(\phi)\right) \tag{10}$$

The total grid emissions per cell (kgCO_2-eq), in a given year is $\xi_{G,n}^{tot}(\phi)$, is the extension infrastructure emissions $\xi_{G,n}^{ext}(\phi)$ added to the $\xi_{G,n}^{Gen}(\phi)$ generation emissions. The model stores a matrix of the emissions occurring in each cell, and each year of the simulation, used for the calculation of carbon taxation amounts. Additionally, it sums emissions over all years in each cell to provide a column vector of total emissions from the grid in each cell, $\xi_G^{tot}(\Phi)$ (in (kgCO_2-eq).

Off-grid Costs

For off-grid systems, system costs and emissions can be defined at the region, country or cell level. For the use case of the model described in this paper, cost and emissions outputs were provided by the CLOVER model at the country level for different off-grid system types. The following description will assume off-grid system inputs at the country level, however; these could vary at the individual cell level. The model is configured to compare off-grid systems powered by diesel or by solar PV; at the household (stand-alone-system) and community level (mini-grids) with national grids. It could be easily adapted to compare additional off-grid means of access.

Although mini-grids and stand-alone systems each powered by PV or diesel will have their system-specific per-household investment cost inputs, the process for calculating respective investment costs for all off-grid systems is the same, and here each of the off-grid systems is given as a type, j. The total costs for an off-grid system C_{og}, of type j in a given map cell $C_{og}^{j}(\phi)$ is defined by the country level investment, $C_{og}^{I}(\kappa)$ and OPEX values, $C_{og}^{O}(\kappa)$; and the number of households added in a given cell $H(\phi)$ in each year n of the simulation; discounted respectively. For a given year n, cell ϕ and off-grid type j, the investment cost (in 2022 USD) is defined by

$$C_{og,n}^{I,j}(\phi) = \left(C_{og}^{I,j}(\kappa)\right)\left(H_n(\phi)\right) \tag{11}$$

For operational expenditure (OPEX) (in 2022 USD) e.g. fuel or new components, the per-household amount for the off-grid type j, is simultaneously factored in for years after the construction year n, up to the final year N. In the model, the OPEX costs are added in and discounted to be in the present value of the year of construction n:

$$C_{og,n}^{O,j}(\phi) = \sum_{x=n}^{N} \frac{\left(C_{og}^{O,j}(\kappa)\right)\left(H_n(\phi)\right)}{(1+r)^{x-n}} \tag{12}$$

where x is any year between the construction year n, and the end of the simulation N. The total cost in a given cell and for an off-grid type, $C_{og}^{j}(\phi)$ is the sum of the OPEX costs and investment costs occurring in all years and discounted to give the present value,

$$C_{og}^{j}(\phi) = \sum_{n=1}^{N} \frac{C_{og,n}^{I,j} + C_{og,n}^{O,j}(\phi)}{(1+r)^{n}} \tag{13}$$

The model stores the costs for each cell ϕ and off-grid type j summed across all years as column vectors, $C_{og}^{j}(\Phi)$, to be used to assess the least-cost mode for each map cell.

Off-grid Emissions

Off-grid emissions follow a similar pattern to costs, explained above, without discounting. However, emissions are tracked on an annual basis, as this is necessary for carbon pricing, which is explained further below. The infrastructure emissions, embedded in the initial capacity installed ξ_{og}^{inf} ($kgCO_2$-eq/kWp/kW or kWh), for a given cell ϕ, year,n and off-grid type, j is

given by

$$\xi_{og,n}^{inf,j}(\phi) = \left(\xi_{og,n}^{inf,j}(\kappa)\right)(H_n(\phi)) \tag{14}$$

where $og^{inf,j}(\kappa)$ is the infrastructure emissions per household in the given country κ and for the off-grid type, j. The OPEX emissions for the given year n, are given by

$$\xi_{og,n}^{O,j}(\phi) = \left(\xi_{og}^{O,j}(\kappa)\right)\left(H_n^{tot}(\phi)\right) \tag{15}$$

whereby H^{tot} is the total accumulated number of households connected in the grid cell in the given year n. The total emissions for an off-grid system type, ξ_{og}^{j} in a cell, ϕ for a specific year, n is the infrastructure emissions $\xi_{og,n}^{inf,j}(\phi)$ added to the $\xi_{og,n}^{O,j}(\phi)$ OPEX emissions. Due to PV systems having a long asset lifetime and the balance of emissions being on the infrastructure rather than fuel inputs, emissions for PV are divided by the asset lifetime (α^{PV}, see Supplementary Table 4) before being factored in for each relevant year between the construction year and the end of the simulation. The output of this process is a matrix of cells covering the geographic area, and years, with respective emissions occurring in each cell in each year of the simulation. This is used for calculating carbon tax (see below).

Inclusion of Carbon Tax

The model can accommodate a single carbon price, or dynamic carbon pricing, varying by year. For this paper, the carbon price varies by year. To calculate the carbon tax values for a given electrification mode, m, and in each cell, ϕ, the model uses the matrices of emissions in each cell, produced in each year (as outlined above). For a given year, n, there is a column vector of cells Φ from the respective matrix containing emissions ξ (kgCO_2-eq), from each electrification mode m. The carbon tax value for the given year as defined by the user, C_n^{tax}, (in 2022 USD / tonne) is multiplied by the emissions from the relevant mode, m, and discounted. The sum of this across all years gives the total carbon tax amount for the mode, $C^{tax,m}(\Phi)$:

$$C^{tax,m}(\Phi) = \sum_{n=1}^{N} \frac{(C_n^{tax})\,\xi_n^m(\Phi)}{(1+r)^n} \tag{16}$$

The net present value of the carbon tax amount, in a column vector of all, $C^{tax,m}(\Phi)$, is included in total costs for each mode, m, for scenarios with a carbon tax.

Inclusion of Unmet Demand Penalty

The model can apply an unmet demand penalty (in $ per kWh unmet) across all electrification modes, m, with the level varying between modes e.g. off-grid versus grid; and in each country, κ. It does so using the reliability, R (% of demand met) for each mode m in each country κ, and the energy demand, E^L (in kWh) in the relevant year n, and cell ϕ such that

$$C^{rel}(\phi) = \sum_{n=1}^{N} \frac{C^{rel}\,(1-R^m(\kappa))\left(E_n^D(\phi)\right)}{(1+r)^n} \tag{17}$$

where the C^{rel} is the financial value of the unmet demand penalty. The total amounts per cell, $C^{rel}(\phi)$, form a column vector $C^{rel}(\Phi)$ to be added to total costs for each mode m, for scenarios including an unmet demand penalty.

Least-Cost Electrification by cell

Once total net present costs, in each cell for all years and for each mode m are calculated, the model then selects the least-cost option for each grid cell based on the total net present cost, factoring in either carbon pricing or unmet demand penalties if specified for the scenario. Prior to this, it uses the population density (persons per cell) to determine whether each cell is most suitable for off-grid electrification. There is a user-defined population density threshold P^{Thr}, below which, for the given cell, the model will compare the total cost for stand-alone-systems, $C_{SA}(\phi)$, in favour of the cost of mini-grid systems, $C_{MG}(\phi)$. This is the case for both PV and diesel-powered off-grid systems, such that

$$C_{og}(\phi) = \begin{cases} C_{MG}(\phi) & \text{if } P_N^{Den}(\phi) > P^{Thr} \\ C_{SA}(\phi) & \text{otherwise} \end{cases} \tag{18}$$

where $P_N^{Den}(\phi)$ is the population density of people getting access in each cell, in the final year of the simulation, N, and $C_{og}(\phi)$ is the selected off-grid system type (stand-alone or mini-grid), based on the population density in the cell. Once establishing whether a cell, ϕ is best suited to stand-alone systems or mini-grids, the next step is to compare the costs off-grid modes of each generation type, PV, C_{og}^{PV}, and diesel, C_{og}^{D} for each cell with the cost of the national grid, C_G:

$$C(\phi) = \min\left\{C_{og}^{PV}(\phi), C_{og}^{D}, C_G(\phi)\right\} \tag{19}$$

Model Outputs

The model outputs a column vector and the cost and respective selected electrification mode for each cell. Outputs from the model are provided in a by-cell output, with data such as emissions, cost and electricity access mode along with the cell location data used for mapping outputs; total scenario appraisal outputs giving the percentage of the population met by each mode and investment and emissions via each mode, and the same appraisal but by-country included in the respective study.

References

1. S. Pfenninger and I. Staffell, *Long-term patterns of European PV output using 30 years of validated hourly reanalysis and satellite data*, Energy, vol. 114, pp. 1251–1265, 2016. https://doi.org/10.1016/j.energy.2016.08.060

2. Google Developer Tools. (2022). *countries.csv | Dataset Publishing Language | Google Developers*. Retrieved from https://developers.google.com/public-data/docs/canonical/countries_csv

3. DNV GL. (2017). *Battery Energy Storage Study for the 2017 IRP*. Battery Energy Storage Study for the 2017 IRP, 40.

4. IRENA. (2017). *Electricity storage and renewables: Costs and markets to 2030*. October, 132. Retrieved from http://irena.org/publications/2017/Oct/Electricity-storage-and-renewables-costs-and-markets. ISBN 978-92-9260-038-9 (PDF)

5. Ioakimidis, C. S., Murillo-Marrodán, A., Bagheri, A., Thomas, D., & Genikomsakis, K. N. (2019). *Life cycle assessment of a lithium iron phosphate (LFP) electric vehicle battery in second life application scenarios*. Sustainability (Switzerland), 11(9), 2527. doi: 10.3390/su11092527

6. Pang, H., Lo, E., & Pong, B. (2006). *DC Electrical Distribution Systems in Buildings*. In *2006 2nd International Conference on Power Electronics Systems and Applications* (pp. 115-119). IEEE. doi: 10.1109/PESA.2006.343082

7. Starke, M., Tolbert, L. M., & Ozpineci, B. (2008). *AC vs. DC distribution: A loss comparison*. In *2008 IEEE/PES Transmission and Distribution Conference and Exposition* (pp. 1-7). IEEE. doi: 10.1109/TDC.2008.4517256

8. Mikhaylov, K., Tervonen, J., & Fadeev, D. (2012). *Development of Energy Efficiency Aware Applications Using Commercial Low Power Embedded Systems*. InTechOpen. doi: 10.5772/38171

9. Tongia, R. (2018). *Microgrids in India: Myths, misunderstandings, and the need for proper accounting*. Brookings Institution. Available from https://www.brookings.edu/research/microgrids-in-india-myths-misunderstandings-and-the-need-for-proper-accounting/

10. MeshPower. (2021). *Private Correspondence with MeshPower Limited*. Personal communication.

11. Elementa and Willmott Dixon. (2022). *Whole life carbon of photovoltaic installations - Technical Report - February 2022*. Retrieved from https://www.willmottdixon.co.uk/asset/17094

12. de Wild-Scholten, M.J. (Mariska). (2013). *Energy payback time and carbon footprint of commercial photovoltaic systems*. Solar Energy Materials and Solar Cells, 119, 296-305. doi: 10.1016/J.SOLMAT.2013.08.037

13. Peters, J. F., Baumann, M., Zimmermann, B., Braun, J., & Weil, M. (2017). *The environmental impact of Li-Ion batteries and the role of key parameters – A review*. Renewable and Sustainable Energy Reviews, 67, 491-506. doi: 10.1016/j.rser.2016.08.039

14. Schmidt, O., Hawkes, A., Gambhir, A., & Staffell, I. (2017). *The future cost of electrical energy storage based on experience rates*. Nature Energy, 2(8), 17110. doi: 10.1038/nenergy.2017.110

15. Färber, R., Guillod, T., Krismer, F., Kolar, J. W., & Franck, C. M. (2019). *Endurance of polymeric insulation foil exposed to DC-biased medium-frequency rectangular pulse voltage stress*. Energies, 13(1), 8. doi: 10.3390/en13010008

16. Smith, C., Burrows, J., Scheier, E., Young, A., Smith, J., Young, T., & Gheewala, S. H. (2015). *Comparative Life Cycle Assessment of a Thai Island's diesel/PV/wind hybrid microgrid*. Renewable Energy, 80, 85-100. doi: 10.1016/j.renene.2015.01.003

17. Institute for European Environmental Policy (IEEP). (2009). *Environmentally Harmful Subsidies: Identification and Assessment' - Annex 5: Subsidy level indicators for the case studies*. Retrieved from http://ec.europa.eu/energy/publications/statistics/doc/2009_energy_transport_figures.pdf

18. Beath, H., Hauser, M., Sandwell, P., Gambhir, A., Few, S., Chambon, C. L., & Nelson, J. (2021). *The cost and emissions advantages of incorporating anchor loads into solar mini-grids in India*. Renewable and Sustainable Energy Transition, 1, 100003. doi: 10.1016/J.RSET.2021.100003

19. Rocky Mountain Institute. (2020). *Electrifying Economies: Comparing Costs of an Integrated Approach versus Pure Grid Extension*.

20. The World Bank. (2022). *World Bank Open Data | Data*. Retrieved from https://data.worldbank.org/

21. The World Bank. (2022). *Enterprise Surveys Indicators Data - World Bank Group*. Retrieved from https://www.enterprisesurveys.org/en/data

22. Global Data Lab. (2022). *Average household size - Area Database - Table - Global Data Lab*. Retrieved from https://globaldatalab.org/areadata/table/hhsize/

23. Allington, L. (2022). *Selected 'Starter kit' energy system modelling data for selected countries in Africa, East Asia, and South America*. doi: 10.21203/rs.3.rs-1178306/v1

24. UNFCCC. (2019). *Harmonized Grid Emission factor data set | UNFCCC*. Retrieved from https://unfccc.int/documents/198197

25. Takahashi, K., & Louhisuo, M. (2022). *IGES List of Grid Emission Factors*. Institute for Global Environmental Strategies, 11.0. Retrieved from https://www.iges.or.jp/en/pub/list-grid-emission-factor/en. doi: 10.57405/IGES-1215

26. Brander, M., Sood, A., Wylie, C., Haughton, A., & Lovell, J. (2011). *Electricity-specific emission factors for grid electricity*.

27. Beath, H - hamishbeath/LEAF-geospatial-energy-africa: Beta-v0.2 (Beta-v0.2) [MODEL] (2024) Retrieved from https://github.com/hamishbeath/LEAF-geospatial-energy-africa.
