# Supplementary material for: Carbon pricing and system reliability impacts on pathways to universal electricity access in Africa
Source: Nat Commun. 2024 May 16;15:4172. doi: 10.1038/s41467-024-48450-7 (PMC11099103; doi:10.1038/s41467-024-48450-7)
Supplement: Supplementary file 3 — Reporting Summary [file 41467_2024_48450_MOESM3_ESM.pdf]

nature portfolio

Corresponding author(s): Hamish Beath

Last updated by author(s): Mar 7, 2024

Reporting Summary

Nature Portfolio wishes to improve the reproducibility of the work that we publish. This form provides structure for consistency and transparency in reporting. For further information on Nature Portfolio policies, see our Editorial Policies and the Editorial Policy Checklist.

Statistics

For all statistical analyses, confirm that the following items are present in the figure legend, table legend, main text, or Methods section.

n/a	Confirmed	
☒	☐	The exact sample size (*n*) for each experimental group/condition, given as a discrete number and unit of measurement
☒	☐	A statement on whether measurements were taken from distinct samples or whether the same sample was measured repeatedly
☒	☐	The statistical test(s) used AND whether they are one- or two-sided<br>*Only common tests should be described solely by name; describe more complex techniques in the Methods section.*
☒	☐	A description of all covariates tested
☒	☐	A description of any assumptions or corrections, such as tests of normality and adjustment for multiple comparisons
☒	☐	A full description of the statistical parameters including central tendency (e.g. means) or other basic estimates (e.g. regression coefficient) AND variation (e.g. standard deviation) or associated estimates of uncertainty (e.g. confidence intervals)
☒	☐	For null hypothesis testing, the test statistic (e.g. *F*, *t*, *r*) with confidence intervals, effect sizes, degrees of freedom and *P* value noted<br>*Give P values as exact values whenever suitable.*
☒	☐	For Bayesian analysis, information on the choice of priors and Markov chain Monte Carlo settings
☒	☐	For hierarchical and complex designs, identification of the appropriate level for tests and full reporting of outcomes
☒	☐	Estimates of effect sizes (e.g. Cohen's *d*, Pearson's *r*), indicating how they were calculated

Our web collection on statistics for biologists contains articles on many of the points above.

Software and code

Policy information about availability of computer code

Data collection	No software was used to collect data in this study.
Data analysis	Code to perform data analysis was written for this study and is available on Github, stated on the manuscript. at hamishbeath/LEAF-geospatial-energy-africa and https://github.com/CLOVER-energy/CLOVER

For manuscripts utilizing custom algorithms or software that are central to the research but not yet described in published literature, software must be made available to editors and reviewers. We strongly encourage code deposition in a community repository (e.g. GitHub). See the Nature Portfolio guidelines for submitting code & software for further information.

Data

Policy information about availability of data

All manuscripts must include a data availability statement. This statement should provide the following information, where applicable:

- Accession codes, unique identifiers, or web links for publicly available datasets
- A description of any restrictions on data availability
- For clinical datasets or third party data, please ensure that the statement adheres to our policy

All data used for running the scenario analysis and for producing figures can be found at the Github repository along with the code for the geospatial tool, at github.com/hamishbeath/LEAF-geospatial-energy-africa. The data (with the code) is archived with Zenodo https://doi.org/10.5281/zenodo.10793959.

Research involving human participants, their data, or biological material

Policy information about studies with human participants or human data. See also policy information about sex, gender (identity/presentation), and sexual orientation and race, ethnicity and racism.

Reporting on sex and gender	N/A
Reporting on race, ethnicity, or other socially relevant groupings	N/A
Population characteristics	N/A
Recruitment	N/A
Ethics oversight	N/A

Note that full information on the approval of the study protocol must also be provided in the manuscript.

Field-specific reporting

Please select the one below that is the best fit for your research. If you are not sure, read the appropriate sections before making your selection.

☐ Life sciences ☐ Behavioural & social sciences ☒ Ecological, evolutionary & environmental sciences

For a reference copy of the document with all sections, see nature.com/documents/nr-reporting-summary-flat.pdf

Ecological, evolutionary & environmental sciences study design

All studies must disclose on these points even when the disclosure is negative.

Study description	The study uses energy system modelling, energy demand modelling and geospatial analysis to explore least-cost pathways to universal household electricity access in Sub-Saharan Africa. The study focuses on the impact of electricity demand, system reliability and carbon pricing on technology shares.
Research sample	Datasets used include those describing the spatial distribution of population and expected population growth, existence of electricity access and infrastructure, technology cost and emissions data, and country-specific data such as household size.
Sampling strategy	Countries included within the study were in sub-Saharan Africa, that do not have full electricity access and for which adequate data to conduct the study was available. Technologies included are those that are most commonly used to address electricity access challenges. This work only looks at the assumed population without access in these countries as the topic is focused on pathways to universal household electricity access.
Data collection	The data used for this study were collected from a variety of publically available sources from existing research or from online repositories.. All data used is referenced and explained within the manuscript.
Timing and spatial scale	For the energy demand and energy system modelling, an hourly temporal scale was used. For the least cost electricity access scenarios, an annual temporal scale was used. For the spatial analysis, all geospatial data layers were aggregated to a 10km squared resolution. The geospatial model also uses a 10km squared resolution for the scenario analysis.
Data exclusions	No data were excluded from the analysis.
Reproducibility	Code and data for scenario analysis is available on Github. All other data is available on reasonable request.
Randomization	Randomisation was not relevant for this study
Blinding	Blinding was not relevant to this study, which relied on computer simulation using existing data inputs.

Did the study involve field work? ☐ Yes ☒ No

Reporting for specific materials, systems and methods

We require information from authors about some types of materials, experimental systems and methods used in many studies. Here, indicate whether each material, system or method listed is relevant to your study. If you are not sure if a list item applies to your research, read the appropriate section before selecting a response.

Materials & experimental systems

n/a	Involved in the study
☒	☐ Antibodies
☒	☐ Eukaryotic cell lines
☒	☐ Palaeontology and archaeology
☒	☐ Animals and other organisms
☒	☐ Clinical data
☒	☐ Dual use research of concern
☒	☐ Plants

Methods

n/a	Involved in the study
☒	☐ ChIP-seq
☒	☐ Flow cytometry
☒	☐ MRI-based neuroimaging

Plants

Seed stocks	N/A
Novel plant genotypes	N/A
Authentication	N/A
